# Management of Patients with Pancreatic Ductal Adenocarcinoma in the Real-Life Setting: Lessons from the French National Hospital Database

**DOI:** 10.3390/cancers13143515

**Published:** 2021-07-14

**Authors:** Christelle de la Fouchardière, Mustapha Adham, Anne-Marie Marion-Audibert, Antoine Duclos, Claude Darcha, Olivier Berthelet, Valérie Hervieu, Pascal Artru, Hélène Labrosse, Yohan Fayet, Bruno Ferroud-Plattet, Bruno Aublet-Cuvellier, Georges Chambon, Mathieu Baconnier, Christine Rebischung, Fadila Farsi, Isabelle Ray-Coquard, Charles Mastier, Pierre-Jean Ternamian, Nicolas Williet, Emmanuel Buc, Thomas Walter, Andrée-Laure Herr

**Affiliations:** 1 Medical Oncology Department, Centre Léon Bérard, 28 Rue Laennec, 69008 Lyon, France; 2 Surgery Department, Hopital Edouard Herriot, 5 Place d’Arsonval, 69003 Lyon, France; mustapha.adham@chu-lyon.fr; 3 Gastroenterology and Hepatology, Clinique du Val d’Ouest, 39 Chemin de la Vernique, 69130 Ecully, France; doc.amma@wanadoo.fr; 4 Research on Healthcare Performance (RESHAPE), INSERM U1290, Université Claude Bernard Lyon 1, 69100 Lyon, France; antoine.duclos@chu-lyon.fr; 5 Pathology Department, CHU Estaing, 1 Rue Lucie et Raymond Aubrac, 63100 Clermont-Ferrand, France; cdarcha@chu-clermontferrand.fr; 6 Hépato-Gastro-Entérologie Department, Centre Hospitalier Métropole Savoie, Place Lucien Biset, 73011 Chambery, France; olivier.berthelet@ch-metropole-savoie.fr; 7 Pathology Department, Hopital Edouard Herriot, 5 Place d’Arsonval, 69003 Lyon, France; valerie.hervieu@chu-lyon.fr; 8 Gastroenterology Department, Hopital privé Jean Mermoz, 55 Avenue Jean Mermoz, 69008 Lyon, France; dr.artru@wanadoo.fr; 9 Réseau Régional de Cancérologie Auvergne-Rhône-Alpes (ONCO AURA), 60 Avenue Rockefeller, 69008 Lyon, France; Helene.LABROSSE@onco-aura.fr (H.L.); Fadila.FARSI@onco-aura.fr (F.F.); 10 Research on Healthcare Performance (RESHAPE), INSERM U1290, Equipe EMS–Département de Sciences Humaines et Sociales, Centre Léon Bérard, 28 Rue Laennec, 69008 Lyon, France; yohan.fayet@lyon.unicancer.fr; 11 Agence Régionale de Santé (ARS) AuRA, 241 Rue Garibaldi, 69003 Lyon, France; Bruno.FERROUD-PLATTET@ars.sante.fr (B.F.-P.); bruno.aublet-cuvelier@ars.sante.fr (B.A.-C.); alherr@lhopitalnordouest.fr (A.-L.H.); 12 URPS Infirmiers Libéraux Auvergne Rhône-Alpes, 21 Quai Antoine Riboud, 69002 Lyon, France; secretariat-general@urps-inf-aura.fr; 13 Hépatogastroentérology Department, Centre Hospitalier Annecy Genevois, 1 Avenue de l’Hôpital, 74370 Epagny Metz-Tessy, France; mbaconnier@ch-annecygenevois.fr; 14 Medical Oncology Department, Medipole de Savoie, 300 Avenue des Massettes, 73190 Challes-les-Eaux, France; c.rebischung@medipole-de-savoie.fr; 15 Research on Healthcare Performance (RESHAPE), EA 7425, Centre Léon Bérard, 28 Rue Laennec, 69008 Lyon, France; isabelle.ray-coquard@lyon.unicancer.fr; 16 Radiology Department, Centre Léon Bérard, 28 Rue Laennec, 69008 Lyon, France; charles.mastier@lyon.unicancer.fr; 17 URPS Médecins Liberaux AuRA, 20 Rue Barrier, 69006 Lyon, France; pjt.urpsaura@gmail.com; 18 Hepatogastroenterology Department, CHU, Avenue Albert Raimond, 42270 Saint-Etienne, France; nicolas.williet@chu-st-etienne.fr; 19 Digestive and Hepatobiliary Surgery Department, CHU Estaing, 1 Rue Lucie et Raymond Aubrac, 63100 Clermont-Ferrand, France; ebuc@chu-clermontferrand.fr; 20 Medical Oncology Department, Pavillons E-UJOMM, Hopital Edouard Herriot, 5 Place d’Arsonval, 69003 Lyon, France; thomas.walter@chu-lyon.fr

**Keywords:** PDAC, pancreatic cancer, adenocarcinoma, real-life data, survival, medical pathway, expertise, disparities, access to care, pancreatic surgery

## Abstract

**Simple Summary:**

With an increasing incidence, late diagnosis, and high mortality rate, pancreatic adenocarcinoma remains, in 2021, a real challenge for health institutions and professionals worldwide. Despite significant therapeutic progress, the five-year survival after diagnosis remains poor. Furthermore, disparities in the access to care (often participating in late diagnosis) have been described in demographic, clinical, and/or socioeconomic factors. We decided to assess in our region, over 1 year (2016), the real-life pancreatic cancer’s management by the analysis of French hospital discharge summaries database system. We pointed out, in a large patient population (*n* = 1872), the inverse correlation between the level of expertise of the health facility in which the patient had his first hospital stay and the likelihood of undergoing any specific treatment for PDAC. A deeper analysis of the medical pathway for pancreatic adenocarcinomas patients is ongoing in order to suggest adapted public health measures.

**Abstract:**

Pancreatic ductal adenocarcinoma (PDAC) remains a major public health challenge, and faces disparities and delays in the diagnosis and access to care. Our purposes were to describe the medical path of PDAC patients in the real-life setting and evaluate the overall survival at 1 year. We used the national hospital discharge summaries database system to analyze the management of patients with newly diagnosed PDAC over the year 2016 in Auvergne-Rhône-Alpes region (AuRA) (France). A total of 1872 patients met inclusion criteria corresponding to an incidence of 22.6 per 100,000 person-year. Within the follow-up period, 353 (18.9%) were operated with a curative intent, 743 (39.7%) underwent chemo- and/or radiotherapy, and 776 (41.4%) did not receive any of these treatments. Less than half of patients were operated in a high-volume center, defined by more than 20 PDAC resections performed annually, mainly university hospitals. The 1-year survival rate was 47% in the overall population. This study highlights that a significant number of patients with PDAC are still operated in low-volume centers or do not receive any specific oncological treatment. A detailed analysis of the medical pathways is necessary in order to identify the medical and territorial determinants and their impact on the patient’s outcome.

## 1. Introduction

With 18.1 million new cancer cases and 9.6 million cancer deaths worldwide in 2018, pancreatic ductal adenocarcinoma (PDAC) represents 2.5% of cancers’ incidence and 4.5% of cancer deaths, according to the International Agency for Research on Cancer (IARC) (GLOBOCAN2018) [1]. In France, in 2018, its incidence has been estimated to be 13,967 new cases (10th rank for the incidence) for a mortality of 13,287 cases (4th rank for mortality). Despite substantial advances demonstrated in phase III clinical trials in the last decade, either in metastatic or in adjuvant settings, the PDAC overall survival remains low, with a 5-year survival rate of less than 10% [2,3,4,5,6]. PDAC mortality will soon exceed breast cancer mortality, explaining its growing burden in terms of public health [7,8]. One factor of crucial progress could be in the early diagnosis of this cancer, but there has been no biomarker validated so far for the screening of this cancer. Pending more significant advances in this field, the optimization of the access to care could likely improve survivals outcomes. Significant disparities in the access to care for PDAC patients have been described. Related factors to these disparities seem to be demographic (age, gender, race/ethnicity, education level), clinical (overweight/obesity, smoking status), and/or socioeconomic (marital status, geographic location, income, insurance status) [9,10,11,12]. However, data are mainly issued from American registries. In France, few epidemiologic studies described the usual medical path for PDAC patients and the related factors. In 2017, Maire et al. described data issued from the French national hospital discharge summaries database system (Programme de Médicalisation des Systèmes d’Information: PMSI). They confirmed an increase of the national incidence between 2010 and 2014, as observed worldwide, and revealed a disparity in the access to care throughout the different regions of France and within the regions themselves [13]. Moreover, hospital-related characteristics (hospital facility type and hospital/surgeon volume) could impact on overall survival in patients with localized PDAC [14,15,16]. In this context, we questioned how PDAC patients are managed in the Auvergne-Rhône-Alpes region (AuRA), France. AuRA is the second-most-inhabited region, after Ile-de-France, and represents approximately 10% of the metropolitan French population.

The primary objectives of the study were to report the PDAC incidence in AuRA over 2016, describe patients’ characteristics according to the hospital expertise, and the type of treatment provided. Secondary objectives were to assess overall survival at 1 year and to describe PDAC patients’ path from the 30 days before the first hospital stay.

## 2. Materials and Methods

This is a retrospective analysis performed in collaboration with the Agence Régionale de Santé (ARS) of AuRA. The national hospital discharge summaries database system (PMSI) was used to analyze the management of patients with newly diagnosed PDAC over the year 2016 in the AuRA region. The PMSI database contains main information about each patient and their inpatient stay, including diagnostic procedures and treatment. Additionally, the status of the unit of care (intensive vs. non-intensive) is reported and was used to define Medicare diagnosis-related groups (DRGs). Every patient firstly hospitalized in AuRA over the year 2016 for a new diagnosis of “pancreatic malignant neoplasm” was identified in the PMSI database using the French version of the International Classification of Diseases (ICD-10). Because of differences in terms of prognosis and treatment, we excluded the C25.4 code corresponding to malignant tumors of the endocrine pancreas. Patients under 18 years old, not residing in France, or who were previously hospitalized for pancreatic cancer during the last two years were excluded. An anonymized identifying code enabled the elimination of potential duplicates. The following data were collected: type of hospital (private, university, general) via the national entity identifier, some patients’ information (age, gender, residence code), and the “concurrent diagnoses” (combining the “primary diagnosis”, defined as the condition that led to hospitalization, the “related diagnosis”, as any underlying condition that may have been related to the primary diagnosis, and the “significant associated diagnosis”, such as complications and comorbidities). We also recorded the need of intensive care, provenance of the patient (from home, emergency care, other hospital), and destination at discharge (other medical unit of the same hospital, another hospital, hospice, palliative care unit, home, or death). The date of death was obtained from the national death registry maintained by the National Institute for Statistics and Economic Studies (INSEE) until 3rd June 2019. Patients’ anonymity was ensured by the unique personal identification number (NIR), defined by 2 successive hash scrambling operations previously described [17]. Hospital sites were categorized into 3 levels of expertise for initial diagnosis and treatment decisions, defined by the following criteria: authorization for chemotherapy administration, authorization for gastro-intestinal (GI) cancer surgery with a procedural volume ≥30/year, high number of endoscopic (≥100/year) or radiologic (≥200/year) biliary drainage procedures, and presence of CT/MRI scan on site. The level was graded as follows: grade 1, if none of the criteria was met; grade 2, if only some of them were met; grade 3, if all criteria were met. The medical path from the 30 days preceding hospital care management was accessible via the national health insurance database. Thereby, it was possible to identify whether patients had general practitioner consultations, gastroenterologist consultations, GI imaging exam, or emergency care visit during this period.

Analyses of the hospital stays, patients’ characteristics, and management were only descriptive. Categorical variables were reported as percentages. Continuous variables were summarized as mean or medians (minimum–maximum). Incidence rates of PDAC (per 100,000 adults) were calculated by dividing the annual counts of new diagnosed PDAC by the annual adult French population size, provided by the INSEE. Overall survival was defined from the date of the first inpatient stay to the date of death or to the time of last follow-up. The 1-year OS rate was calculated by dividing the number of nondeclared as dead patients by the total patient population.

## 3. Results

This section may be divided by subheadings. It should provide a concise and precise description of the experimental results, their interpretation, as well as the experimental conclusions that can be drawn.

### 3.1. Population, Structures, and Incidence

**Figure 1:**
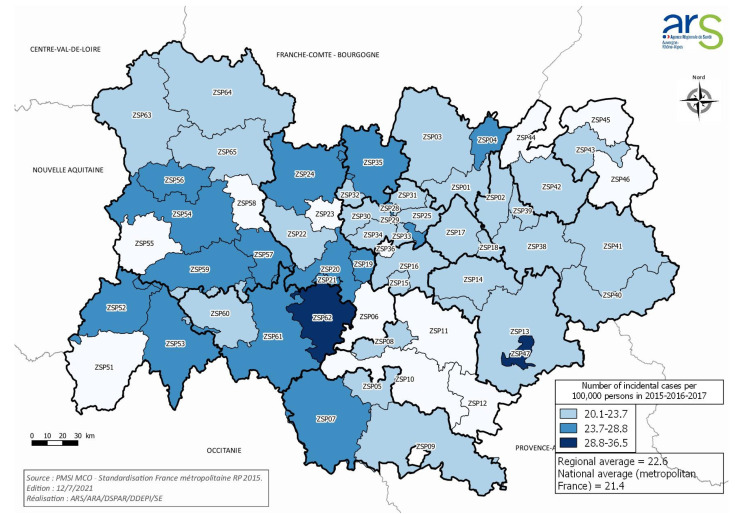
Pancreatic ductal adenocarcinoma (PDAC) in Auvergne-Rhône-Alpes, France in 2016: average annual incidence rates standardized by region of residence.

A total of 1872 patients met the inclusion criteria. They were hospitalized in 118 different hospital structures in 2016, 19 identified as belonging to the level 1 expertise, 87 to the level 2, and 12 to the level 3 (Table 1). The incidence of PDAC in the AuRA region was 22.6 per 100,000 person-year (PY). The highest standardized incidence rates concerned two areas in AuRA (Yssingeaux; La Mure, France), with an incidence of 28.8 and 36.5 cases per 100,000 PY, respectively (Figure 1).

### 3.2. Patient and Treatment Characteristics

The mean age of the entire cohort was 72 years (range: 20 to 101), 51.6% were men, and 619 (33%) had metastases identified during the first hospital stay. A total of 368 patients (19.6%) were declared suffering from malnutrition, 320 patients (17%) had known diabetes, and 41 (2%) were declared obese (BMI ≥ 30). Alcohol consumption was stated in 51 patients and tobacco use in 54 of them. We separated the entire population into three groups of patients according to their therapeutic management: patients operated with a curative intent (group A), patients treated with chemo- and/or radiotherapy (group B), and those who underwent only best supportive care or any declared specific treatment (group C).

A total of 353 patients (group A) were operated in 42 different hospital sites, with a median delay of 43 days from day 1 of their first hospital admission (0–347). The mean age of the patients in this subgroup was 67 years (range: 23 to 90). Less than half of them were operated in university hospitals (*n* = 142, 40%), 123 (35%) in private clinics, and 42 (12%) in general hospital (GH), the rest of them being operated in private hospitals committed to public service, cancer care centers, or outside the region (Table 2). According to our criteria for hospital expertise, 229 patients (66%) were operated in a level 3 institution, whereas none were operated in a level 1 facility. The mean volume of pancreatic resections (PR) for PDAC was 8 (range 1–44) per year (PY). After the exclusion of seven patients operated out of the AuRA region, 154 (44.5%) were operated in a center performing more than 20 PR for PDAC PY, whereas 94 (27%) were operated in centers performing less than 10 PR PY (Table 3). Within this group of 353 patients who underwent PR, 43 patients (12.2%) were declared as having metastatic cancer, without any other precision about the metastasis diagnosis circumstances. A total of 161 patients (45.6%) had upfront surgery, 43 (12.2%) received neoadjuvant chemo- and/or radiotherapy, and 149 (42.2%) of them had another reason for admission to the hospital. An endoscopic biliary drainage was realized for 180 patients (51%) before surgery. Fifty-one (14%) patients were admitted to the intensive care unit during the postoperative period, and 17 (5%) died within the 90 days after the surgery.

Among the 743 patients who underwent only medical treatment (group B), 665 (36%) received chemotherapy, 8 received radiotherapy, and 70 patients received both. Their mean age was 69 years ± SD (range: 29 to 93).

In Group C, there was a total of 776 patients, (mean age: 77 years; range: 20 to 101) who received neither surgery, nor chemotherapy or radiotherapy for their PDAC. Among them, 358 (46%) patients were noted as having received palliative care as a “significant associated diagnosis” in at least one of their hospital stays. For the 418 remaining patients, no information on a specific medical or palliative treatment was available.

The likelihood of undergoing any specific treatment for PDAC increased with the level of expertise of the health facility in which the patient had his first hospital stay (Table 4). Indeed, a pancreatic resection was realized in 1 of the 27 patients admitted to a level 1 hospital (3.7%) versus 168 of 1094 (15.4%) patients who accessed initial care in a level 2 facility, and 184/751 (24.5%) in a level 3 institution (χ^2^ goodness of fit *p* < 0.0001). Likewise, chemo- and/or radiotherapy was offered in 5 of 27 (18.5%) patients in the level 1 hospital sites, versus 415/1094 (37.9%) in level 2, and 323/751 (43%) in level 3 institutions (χ^2^ goodness of fit *p* < 0.0001).

### 3.3. Management within the 30 Days before the First Hospital Stay for PDAC

During the 30 days before the first hospital stay, 1202 patients (64%) were seen by their general practitioner, 299 (16%) by a gastroenterologist, and 172 (9%) were admitted and discharged from the emergency department (34%). Few of them had an abdominal imaging: ultrasound for 351 patients (19%), CT scan for 233 patients (12%), or an MRI for 107 (6%). In total, 452 patients (24%) were admitted via the emergency unit.

### 3.4. Overall Survival

At the end of the period of study (June 3rd 2019), a total of 1354 deaths (72%) were declared. The 1-year survival rate was 47% in the overall population. The corresponding 1-year survival rates for patients issued from groups A, B, and C were 82, 48, and 31%, respectively. The majority of deaths (84%) occurred in medical institutions: 70% in short-stay hospitals, 6% in rehabilitation units, and 8% in the home care setting. Among the 518 patients for whom any death status was available (28%), 384 patients had at least one healthcare reimbursed by the national insurance (Assurance Maladie) in 2018 and/or 2019, justifying considering them as still alive. The 134 remaining patients who did not have any health care reimbursement during this period were considered as dead, because most of them were issued from group C (*n* = 77; 57%) and B (*n* = 46; 34%). Hence, the total number of deaths increases to 1488 patients (79% of the global population).

## 4. Discussion

The present study reports a high incidence of PDAC (22.6 per 100,000 PY) in AuRA region in 2016, which represents 13% of the total number in France during 2016. Studies in breast, prostate, and colorectal cancers have already showed that incidence estimated from the PMSI database was close to that of registries, which supports the robustness of our data [18,19]. In the present study, several crucial information about the “real-life” management of PDAC patients in AuRA can be extracted.

First, in 2016, the majority (55%) of patients with PDAC were still operated in low- or intermediate-volume centers (defined by < 20 pancreatectomies/year). These results are in accordance with those previously reported by Farges et al. in a large French cohort of 22,366 patients, showing that 53% of pancreatectomies were carried out in centers performing less than 25 a year [20]. Despite the absence of consensual definition of low-volume (LV) versus high-volume (HV) hospitals, numerous studies have demonstrated that both postoperative morbidity and mortality are correlated with the surgical activity volume [21,22,23,24,25]. In a meta-analysis, hospital volume was the only predictor of postoperative mortality and survival. In contrast, surgeon volume did not seem to significantly impact these outcomes [26], which suggests that failure-to-rescue major complications after pancreatic surgery, especially concerned by low to moderate hospital volume, may be one of the major explanations for an increase in postoperative [27], others have suggested that surgery centralization could improve resection rates and long-term survival [28,29,30]. Hence, pancreatic surgery should be performed in only high-volume centers to decrease postoperative morbidity–mortality, as yet recommended for esophageal cancer [31]. Nonetheless, despite all these strong evidences, centralization of pancreatic surgery to high-volume centers has failed to be established in most European countries [32]. It is possible that recourse of the responsibility of health authorities will be needed to prevent pancreatectomies for PDAC in some unsuitable structures.

The second interesting result highlighted by our study was the high proportion (41%) of patients who did not receive any specific oncological treatment for PDAC during the time of follow-up. However, few data were available on these patients, including the reasons for a management (very low performance status, heavy comorbidities, patient’s desire?). In the comparable 2013 American SEER data, PDAC-related deaths occurred early in patients with metastatic disease who did not undergo any specific oncological treatment (50.6% within 2 months after the diagnosis) [33]. As expected, in our study, nontreated patients (group C) were older than the others (group B) (77 vs. 69 yrs, respectively). Finally, we show that the probability of undergoing active cancer treatment (pancreatic surgery or chemo/radiotherapy) increases with the expertise level of the hospital where the patient is initially admitted. This point may be explained by more severe cases being presented by themselves or oriented by primary care services directly to referral centers. However, the difference between treatment orientations of patients initially admitted to level 2 and 3 expertise hospitals also argues for the role of intrinsic hospital expertise in the access to more aggressive, possibly curative, treatment options.

The strengths of our study include the large size of the PMSI database, the prospective data collection, its exhaustivity about hospital resources (private or public hospitals), duration of hospital stays, with interesting information about the medical path within the 30 days before the first hospital stay, and place of deaths. The results may have been limited by the intrinsic characteristics of medico-administrative databases, especially the validity of diagnosis codes, inherent to those of the ICD-10, and also includes the lack of detailed clinical and demographic data, prognostic factors (like ECOG-PS), and results of paraclinical examinations. Some other variables, like overweight/obesity, smoking status, and alcohol consumption, may have been underestimated due to a lack of declaration. Other lacking factors, like education level, ethnic group, marital status, and income, may also have an impact on the access to care of PDAC patients. Furthermore, no uni/multivariate analysis was possible, considering our restricted access to the database and the patients’ data anonymization. In order to continue our work, we initiated an observational study called PANDAURA (CNIL authorization n°919240) to describe precisely at a regional level the patients’ paths from 30 days before the first hospital stay for PDAC until death. Our objectives are to analyze the impact of the different time frames, from diagnosis to treatment initiation, evaluate the adhesion to clinical guidelines, and identify any territorial inequalities in the patients’ path. We also aim to understand the impact of environmental (air pollution, pesticides) and social factors (socio-economic level of the territories) on the risk of occurrence of PDAC. The ultimate goal would be to improve the organization and quality of care for patients with PDAC in the AuRA region and, ultimately, nationwide.

## 5. Conclusions

We showed that one in two PDAC patients was operated in a low- or intermediate-volume center in 2016 in AuRA. Based on all literature findings, this crucial point needs to be handled by health authorities. Moreover, further investigations remain needed regarding the 41% of the patients who never received any oncological treatment for their pancreatic cancer. The initial management of a proportion of them likely could have been optimized. Reasons for the suboptimal management of this patient subgroup should be identified to make improvements.

## Figures and Tables

**Table 1:** Distribution of hospital sites by level of expertise and type of structure with number of patients initially admitted with PDAC.

Institution Type	N Hospital Sites	Level 1	Level 2	Level 3	N patients (%)
General hospital	59	13	42	4	640 (34%)
Private clinic	36	2	30	4	637 (34%)
University hospital	12	1	7	4	435 (23%)
Private hospital committed to public service	9	3	6	0	107 (5.7%)
Cancer care center	2	0	2	0	53 (2.8%)
Total	118	19	87	12	1872 (100%)

**Table 2:** Distribution of patients who underwent curative pancreatic surgery (group A) according to hospital structure type performing the surgical act.

Hospital Type	N Hospital Sites	N Patients	(%)
General hospital	14	42	−12%
Private clinic	20	123	−35%
University hospital	4	142	−40%
Private hospital committed to public service	3	19	−5%
Cancer care center	1	20	−6%
Out of region hospital sites	7	7	−2%
Total	42	353	

**Table 3:** Distribution of operated patients according to the hospital expertise level.

Hospital Expertise Levels	N Hospital Sites	N Patients
1	0	21
2	31	117
3	11	229
Total	42	346 *

* 7 pts were operated out of the AuRA region.

**Table 4:** Distribution of hospital sites and number of patients operated according to hospital pancreatic surgery acts’ reference volume/year.

Number of PS/Year/Hospital Site	N hospital Sites	N Patients	(%)
1	14	14	(4.0%)
2–5	15	49	(14.2%)
6–10	4	31	(9.0%)
11–20	6	98	(28.3%)
>20	3	154	(44.5%)
Total	42	346	

## Data Availability

The data presented in this study are available on request from the corresponding author. The data are not publicly available due to privacy.
